# Female Genital Mutilation/Cutting among Somali Women in the U.S. State of Arizona: Evidence of Treatment Access, Health Service Use and Care Experiences

**DOI:** 10.3390/ijerph18073733

**Published:** 2021-04-02

**Authors:** Georgia Michlig, Nicole Warren, Merry Berhe, Crista Johnson-Agbakwu

**Affiliations:** 1 International Health Department, Johns Hopkins School of Public Health, Baltimore, MD 21205, USA; 2 Johns Hopkins School of Nursing, Baltimore, MD 21205, USA; nwarren3@jhu.edu; 3 University of Arizona College of Medicine—Phoenix, Phoenix, AZ 85004, USA; mberhe@email.arizona.edu (M.B.); cejohn11@asu.edu (C.J.-A.); 4 Southwest Interdisciplinary Research Center, Arizona State University, Tempe, AZ 85281, USA; 5 Refugee Women’s Health Clinic, Obstetrics & Gynecology, Valleywise Health, Phoenix, AZ 85008, USA

**Keywords:** female genital cutting, female genital mutilation, satisfaction in care, health service use, access to care, quality of care

## Abstract

Background. Female genital mutilation/cutting (FGM/C) is associated with adverse sexual, reproductive and psychological sequelae. The aim of this study was to quantitatively explore factors related to satisfaction with FGM/C-related care in the US focusing on access to care, health service utilization, and women’s experiences. Methods. A community-based survey of 879 Ethnic Somali and Somali Bantu women using snowball sampling was conducted in Arizona. Bivariate, multivariable and ordered logistics analyses assessed the relationship between the aforementioned factors measured along six dimensions: non-discrimination, physical, economic, informational, health system accessibility and individual-level health service use factors. Findings. Most participants possessed FGM/C (77.4%), namely Type III (40.2%). FGM/C related health service use was low (14.3%). Perceived discrimination was associated with reduced satisfaction in care (OR = 0.22; CI 0.13–0.37). For FGM/C-specific variables, only recollection of adverse physical or psychological events at the time of circumcision predicted service use (OR = 3.09; CI 1.67–5.68). Somali Bantu (OR = 0.10; CI 0.02–0.44) and highly acculturated women (OR = 0.39; CI 0.17–0.86) had lower odds of service use. Conclusions. Achieving respectful care and outreach to women affected by FGM/C has contextual complexity. However, the clinical implications and insights provided may have broader impacts on advancing health equity for FGM/C-affected women.

## 1. Introduction

Since 1991, ongoing civil war in Somalia has fueled a global diaspora. In 2017, prior to the Trump administration’s restrictions on incoming refugees, there were an estimated 174,122 Somalis in the US, and more than 47,400 Somali refugees arrived between 2010–2016 [1,2]. These migrants include Ethnic Somalis, the ethnic majority group, and Somali Bantu, the largest non-ethnic minority who have been marginalized by a history of slavery and displacement in Somalia [3,4,5]. Due to differences in appearance, language, and culture, Somali Bantus are often considered distinct from Ethnic Somalis; these differences are suspected of having an adverse impact on pre- and post-migratory experiences and health [6].

According to the World Health Organization, Female Genital Mutilation/Cutting (FGM/C) “comprises all procedures that involve partial or total removal of the external female genitalia or injury to the female genital organs for nonmedical reasons” [7]. Somali women have the highest reported rates of FGM/C in the world at nearly 98% prevalence [8]. Historically, Somalis practiced the most severe form of FGM/C, Type III or infibulation, which entails near complete closure of the vaginal opening by cutting and appositioning the labia in order to create a seal, which may or may not be accompanied by the removal of portions of the external clitoris [7]. Recent evidence suggests a shift to less severe cutting [9,10]. FGM/C is associated with increased health risk and complications both in the short and long term [11,12]. Immediate complications can include severe pain, excessive bleeding, infections, problems with urination, shock and even death. Long term urinary complications can include pain with urination and urinary tract infections. Lasting gynecologic complications can include painful menstruation and difficulty passing menstrual blood due to obstruction of the vaginal opening, excessive scar tissue, as well as sexual health problems. Obstetric complications can include prolonged labor and increased risk of cesarean section. Limited evidence suggests potential long term psychological impacts, including increased risk of post-traumatic stress disorder, anxiety disorders, and depression [13].

Care seeking is defined as any action by individuals who perceive themselves to have a health problem or to be ill for the purpose of finding an appropriate remedy [14]. Migrants and specifically Somalis, may have unique care seeking and Health Service Use (HSU) considerations including the context of emigration and FGM/C-specific care [15]. Past research has indicated that migration and acculturation may play important roles in FGM/C-affected women’s attitudes regarding FGM/C as well as their experience of their own health and functioning [16]. The notion of acculturation and its operationalization in research is complex and much debated [17]. Public health operationalizations often draw on the bidimensional model, which first asks whether and to what extent an individual maintains their original cultural identity post-migration, and simultaneously assesses whether and to what extent the individual adopts the new culture to which they are exposed [18].

There is rich qualitative data about Somali women’s health experiences in the US. Recurrent themes include communication and language barriers, provider distrust, feelings of discrimination and vulnerability, female provider preference, poor health literacy, divergent expectations about healthcare in the US, and reservations around cesarean section [9,19,20,21]. These concerning data are focused on individual client-provider interactions and reveal gaps in generalizable knowledge on FGM/C, HSU, access to care, and patient satisfaction.

Somali women’s challenges in the health care system resonate with the current discourse around mistreatment of other childbearing women of color in the US. Somalis, like all women, have a right to Respectful Maternity Care (RMC), which protects childbearing persons’ autonomy, dignity, feelings, choices, and preferences [22]. In an effort to better understand Somali women’s care experiences in the US, a quantitative approach is utilized here to provide data regarding potential gaps in care provision. The aim of this study was to quantitatively explore factors related to satisfaction with FGM/C-related care. Specifically, our aims were to: (1) explore factors influencing access to care, (2) describe patterns of and factors influencing health services utilization (HSU), and (3) describe women’s experience of and ultimately, satisfaction with care.

## 2. Materials and Methods

### 2.1. Conceptual Framework

**Figure 1:**
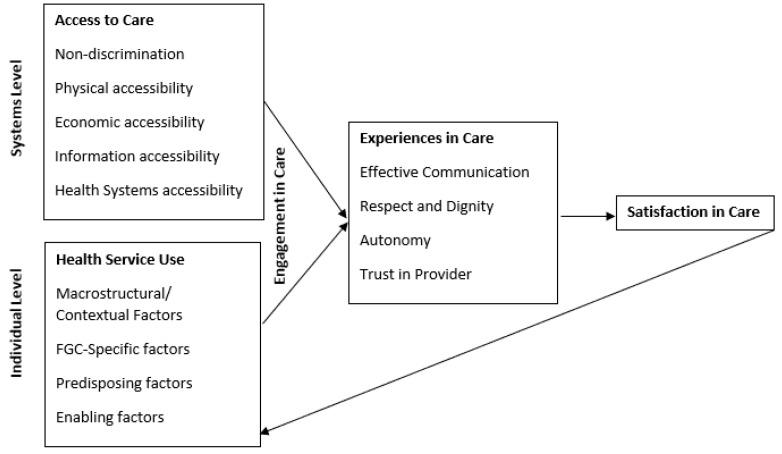
Conceptual model of factors associated with satisfaction in FGC-related care.

Conceptual factors associated with satisfaction in FGM/C-related care were drawn from various social theories to permit a holistic, rights-based view (Figure 1). Access to care was conceptualized according to the four domains delineated by General Comment No. 14 of the United Nations outlining health as a human right [23]. A fifth domain, health system accessibility, was added to address systems-level barriers. Health service use, influenced by Andersen (1995) [24], included macrostructural (the external environment), predisposing (such as demographics and health beliefs), and enabling (social or other resources) factors with an emphasis on FGM/C-related care [15]. Domains relevant to experiences impacting satisfaction in care were informed by the WHO’s Quality of Care Framework for maternal and newborn health [25] and qualitatively derived experiences of mistreatment among women in obstetric care [26]. The organization of the framework first into a separation between individual and systems level factors is aligned also with Andersen’s model of health service use, where systems level factors are meant to represent the overall functioning of the health system with which the individual interacts, while the individual level relates to factors outside of health system functioning that influence engagement in carel as described in Figure 1. It should be noted that some factors, such as discrimination, exist at multiple levels. This framework proposes that these factors combine to create the environment and conditions within which persons experience care, leading to that individual’s eventual satisfaction or dissatisfaction with the care they received. Anderson notes that a feedback loop exists, where a health service utilization outcome such as satisfaction in care will affect future predisposing factors at the individual level [24].

### 2.2. Data Collection and Measures

A community health needs assessment survey (CHNAS) of Somali women (*n* = 879) was conducted in Phoenix and Tucson, Arizona in 2017. This was part of a larger study (ASTWH160045-02-00) designed to improve health services to women affected by FGM/C in Arizona [27]. Incorporating principles of Community-Based Participatory Research (CBPR) is foundational to engaging communities on such a sensitive, stigmatized and taboo-laden topic as FGM/C [28]. The Somali community in Maricopa County, Arizona has had a long track record of CBPR engagement through the community’s partnership with a dedicated Refugee Women’s Health Clinic (RWHC) [28], housed within one of the leading public safety net health care systems in the State, which specializes in the health care of FGM/C-affected populations. In addition, academic partners have nurtured and sustained partnerships grounded in trust, and cultural embeddedness in advancing reproductive health equity within this community. The Refugee Women’s Health Community Advisory Coalition (RWHCAC) comprises refugee-serving community stakeholders encompassing ethnic community-based organizations [29], faith-based organizations, public health, health care [30,31] and social service entities, refugee resettlement agencies, and Community Health Workers (coined ‘Cultural Health Navigators’), who shape and inform the clinical, programmatic and research priorities and direction of the RWHC. As an extension of this longstanding effort, the CHNAS instrument was developed through collaboration between the research team and the RWHCAC through community key informants representing the ethnic, linguistic, gender, and generational diversity of our broader Somali study population. Through an iterative process, these community key informants shaped the development of the CHNAS to achieve cross cultural equivalency and ensure cultural responsiveness. Ethical approval was provided by Arizona State University.

The survey was forward and back-translated from English into the Somali and Maay Maay languages, and subsequently modified by Somali and Somali Bantu community key informants to achieve linguistic and cultural equivalency. Somali women community members were trained as data collectors and administered the survey verbally in the language of choice, after receiving informed consent. Parental consent and child assent were ascertained for minors under the age of eighteen. The survey was quantitative in nature (including numerical, binary, multiple-choice and likert response options) and was 90 questions in length, including specific modules assessing demographics, acculturation (description below), reproductive health, health-seeking behaviors, provider experiences, and knowledge/beliefs/experiences with FGM/C. The Refugee Health Screener 13 was used to screen for signs of clinically significant distress [32]. Surveyors collected data on digital tablets and data management was facilitated by REDCap software [33]. The average time to completion was 1 h.

Participants were eligible if they were at least 15 years old, Ethnic or Bantu Somali ethnicity, identified as female, lived in the Phoenix or Tucson metropolitan areas of Arizona, US. A respondent-driven sampling (RDS) approach was initially attempted; however, after one month of data collection, and due to rising tensions in the community surrounding emerging immigration and refugee policies, the strategy was altered to snowball sampling.

Circumcision status was determined by asking if the woman was circumcised (yes/no) and if yes, to what degree. A visual aid was utilized to help women self-report the type of cutting [34]. Recall of experiencing health problems at the time of circumcision included assessing whether the women recalled extreme pain, excessive bleeding, shock, infection, pain or difficulty with urination, or a prolonged/difficult recovery, and included an “other” write in option. FGM/C HSU was extrapolated as a binary variable from women’s reports of seeking professional health care for various medical concerns and follow up questions assessing if they considered this condition to be related to their FGM/C. Concerns included a list of obstetric (difficulty getting pregnant, infertility, post-term pregnancy, stillbirth, fetal distress, emergency c-section, infant resuscitation, extensive vaginal tears, postpartum hemorrhage, prolonged hospitalization), urinary and gynecologic (pain with menstruation, other recurrent bleeding, difficulty passing urine, pain with urination, recurrent urinary tract infection, vaginal itching, recurrent genital infections, cysts in genital area, scarring in genital area, fistula), sexual (delayed intercourse, inability to have intercourse, pain with intercourse, lack of pleasure during sex, lack of sexual desire, bleeding with intercourse) and mental health (feeling sad for many weeks at a time, having flashbacks or nightmares) complications.

Acculturation was considered a predisposing factor of HSU and was measured using the Modified Bicultural Involvement Questionnaire (M-BIQ). This tool has been previously validated in this community [35,36] and includes four patterns of acculturation [37,38] that we define here as follows: Traditional, strongly oriented towards Somali culture with limited interaction with U.S. culture, Acculturated, strongly oriented towards U.S. culture, with limited interaction with Somali culture, Bicultural, reflecting an integration of the two cultures; and Hypocultural, with limited involvement with either culture.

Experiences of care were explored by examining communication, respect and dignity, autonomy and trust in providers. 

### 2.3. Data Analysis

We used STATA 14 [39] to conduct the analyses. Of the 879 women who participated in the survey, 680 (77.4%) reported being circumcised. Of these, 11.9% were excluded from the analysis because they could not (*n* = 75) or declined (*n* = 7) to self-identify a FGM/C type.

Variables were first explored descriptively. Bivariate logistic regression was used in cases where the dependent variables were dichotomous. Analysis of HSU for FGM/C proceeded utilizing a clustering technique according to the domains of FGM/C HSU outlined in Figure 1. In each cluster (macrostructural, FGM/C-specific, predisposing, and enabling) independent variables associated in bivariate logistic regression with FGM/C HSU at a significance level *p* < 0.05 were included in a subsequent model adjusting for age and other variables of that cluster. Variables were assessed for collinearity. The final model retained those variables independently associated with FGM/C HSU in their clustered model.

In order to better understand demographic characteristics potentially associated with each tier of care experiences impacting satisfaction with care, demographic variables including FGM/C type, age, ethnic subgroup, acculturation and education were fitted as independent variables onto each care experience in multivariate logistic or multivariate ordered logistic modeling. When testing likert response items as dependent variables, ordinal logistic regression was used. The proportional odds assumption was verified in each case based on non-significant results of a likelihood ratio test with a null hypothesis of no difference between models.

## 3. Results

A total of 879 Somali women were surveyed with an average age of thirty-one. The demographic and FGM/C-specific variables of participants are summarized in Table 1. In summary, most were Ethnic Somali refugees who considered themselves to be in excellent or very good health and who had public health insurance. Most were circumcised. (*n* = 680, 77.4%). Of those who were circumcised, and for whom type was provided, the highest rates were either Type I (*n* = 223, 36.8%) or Type III (*n* = 243, 40.2%). FGM/C-affected women infrequently reported seeking care related to their FGM/C (*n* = 98, 14.3%).

### 3.1. Access to Care

Access to care was measured on five dimensions (Table 2). With respect to non-discrimination, 16% of participants (*n* = 136) believed that women with FGM/C were discriminated against by healthcare providers. Perceived discrimination was associated with reduced satisfaction in FGM/C-related care (OR = 0.22; CI 0.13–0.37) and having sought FGM/C-related care in the past (OR = 1.89; CI 1.10–3.26). Three quarters (*n* = 490, 75.2%) of participants reported that they knew where to go to access health care for FGM/C-related health concerns, and this had a positive impact on both HSU (OR = 1.79; CI 1.01–3.16) and satisfaction (OR = 6.32; CI 3.82–10.45). Physical accessibility was rarely reported as a barrier (*n* = 5, 0.7%). Economic accessibility was an uncommon concern, with six participants (0.9%) reporting that they could not afford FGM/C-related care, and 16 (2.4%) reporting that their insurance would not approve their FGM/C-related care. Challenge with insurance approval was associated with dissatisfaction in care (OR = 0.14; CI 0.05–0.41). Women most frequently reported experiencing health-system level barriers, with 24 women reporting they could not receive a referral for FGM/C-specific concerns (3.5%) and 39 (5.7%) stating that they could not find a specialist in FGM/C care. Two women (0.3%) reported that they could not get an appointment. Health-system level barriers negatively impacted satisfaction in care.

### 3.2. FGM/C-Related Health Service Use

FGM/C-related HSU was associated in bivariate analysis with a number of variables at the macrostructural, FGM/C-specific, predisposing and enabling levels of the model (Table 3). However, for FGM/C-specific variables, only recollection of experiencing adverse physical or psychological events at the time of circumcision was predictive of FGM/C HSU (OR = 3.09; CI 1.67–5.68) in the final model. In terms of predisposing factors, duration of time in the US and gender of provider did not influence HSU; however, ethnicity and acculturation did. Somali Bantu had significantly lower odds of FGM/C HSU (OR = 0.10; CI 0.02–0.44) compared to Ethnic Somali. As compared to individuals with patterns of cultural involvement consistent with a Traditional cultural orientation, Acculturated individuals were less likely to seek services (OR = 0.39; CI 0.17–0.86) as were the Hypocultural (OR = 0.27; CI 0.10–0.70). Enabling resources appeared largely insignificant in regard to FGM/C HSU.

### 3.3. Care Experiences and Satisfaction in Care

Overall, participants reported positive FGM/C care experiences (Table 4). Just over half of women reported confidence in advocating for the FGM/C-related care they desired (*n* = 475, 59.4%) and most reported comfort discussing concerns with providers (*n* = 518, 79.1%). As it concerns ensuring the respect and dignity of women seeking FGM/C-related care, most women reported “a great deal” or “a fair amount” of respectful treatment (*n* = 690, 88.6%), and no sense of discrimination (*n* = 709, 83.9%). The majority of participants felt they had autonomous choice in where they received care (*n* = 546, 65.2%), their provider (*n* = 499, 59.8%), and what services they received (*n* = 512, 61.1%). Women also felt they had the freedom to express their desires on how they wanted their FGM/C managed (*n* = 467, 70.6%). Finally, women mostly trusted their providers to give good quality FGM/C-related care (*n* = 589, 73.6%).

A relatively small portion of circumcised women reported dissatisfaction with the quality of healthcare they had received surrounding their circumcision (*n* = 81, 12.7%). Dissatisfaction with care was not significantly associated with FGM/C HSU. In bivariate analysis, all aspects of the care experience, including effective communication, respect and dignity, autonomy, and trust in their provider were significantly associated with satisfaction in care (Table 4).

In multivariate modeling, only ethnic group, acculturation and education were significantly associated with care experience. FGM/C type was not significantly associated with any particular care experience, and increasing age was associated only with less comfort in discussing FGM/C health needs with providers (OR = 0.97; CI 0.95–0.99). Somali Bantu, as compared to Ethnic Somali, had higher odds of reporting they did not feel confident advocating for their FGM/C-related health needs (OR = 3.26; CI 1.99–5.34), reported less comfort discussing FGM/C with their provider (OR = 0.43; CI 0.23–0.78), felt they were treated with less respect and dignity (OR = 2.95; CI 1.78–4.91), felt women with FGM/C were looked down upon by providers (OR = 2.64; CI 1.44–4.86), felt less able to freely express their desires regarding their FGM/C management (OR = 0.26; CI 0.15–0.45), and trusted their provider less to provide quality FGM/C-related care (OR = 0.22; CI 0.12–0.40).

Acculturation as measured by patterns of cultural involvement also appeared relevant to care experience. As opposed to those with Traditional cultural orientation, Acculturated individuals reported higher odds of lacking confidence in ability to advocate (OR = 3.26; CI 1.99–5.34), and lower odds of comfort discussing FGM/C (OR = 0.34; CI 0.18–0.64), trust they would receive quality care (OR = 0.45; CI 0.23–0.90), and feeling they could freely express desires (OR = 0.48; CI 0.26–0.88). These associations and their significance were similar for Bicultural individuals. Acculturated participants had other concerns surrounding autonomous choice, including higher odds of reporting they felt less choice in where they received care (OR = 2.01; CI 1.19–3.39), who they received care from (OR = 1.98; CI 1.19–3.30), and which services they received (OR = 2.28; CI 1.40–3.70). Two unique associations between acculturation and care experiences were that Bicultural individuals alone, as compared to Traditional, had higher odds of reporting being treated without respect or dignity (OR = 2.84; CI 1.59–5.05), and Hypocultural individuals had lower odds of feeling women with FGM/C were looked down upon (OR = 0.25; CI 0.09–0.74).

Having received any formal education increased the odds of comfort discussing FGM/C with providers (OR = 1.76; CI 1.01–3.04), feeling they could freely express their desires (OR = 2.13; CI 1.28–3.57), and trust in the quality of care they would receive (OR = 2.30; CI 1.26–4.20).

## 4. Discussion

The majority of our 879 participants were ethnic Somali refugees with public insurance who had experienced FGM/C. Few reported having sought healthcare for FGM/C. Of those who did, few reported barriers to accessing such care. These access issues were most commonly related to identifying an FGM/C-specialist or obtaining a referral to one. HSU was not influenced by refugee status or FGM/C type. HSU was higher among Somali-Bantu women, women with a history of adverse psychological or physical trauma events at the time of cutting, and traditional cultural orientations. Overall, women reported positive care experiences, including care related to their FGM/C. Somali-Bantu women and Acculturated women had poorer care experiences. Having had any education was associated with improved care experience.

Our first aim was to explore systems-level factors that influence access to care. First, it is important to note that just 14% of participants reported seeking care specific to their FGM/C. Given the severe long-term morbidity commonly reported in the literature, this relatively low rate of FGM/C health care seeking was surprising. There are multiple possible explanations. Among those who did experience barriers to care, inability to pay for the care and difficulty finding the right provider were the most common reasons. The percentage of women reporting barriers who cited an inability to pay for FGM/C-specific care was nearly twice that of the estimate of persons in the US who cannot obtain medical care due to cost (9.5% vs. 4.8%) [40]. This is consistent with our sample’s demographics who are mostly publicly insured and, by definition, with lower incomes. The other main barrier was difficulty finding FGM/C-related care or obtaining a referral to a specialist. This is a telling finding given that a large part of data collection for this study was conducted in a city where one of the authors, a national expert in FGM/C-related clinical care is based, and where she and her team have conducted extensive provider education. If women in this sample are reporting difficulty finding the right provider, this has discouraging implications for women in other regions without such expertise. Beyond Arizona, providers with FGM/C-related expertise are concentrated in a few small centers in the US. Since this survey was conducted, a network of providers with an interest and, in many cases, growing expertise, in FGM/C-related care has been established (https://endfgmnetwork.org/members/, accessed on 31 March 2021). While it is useful that providers can now consult and discuss clients more easily through this network, our data suggest that efforts are needed to ensure affected women can find providers with expertise, or access expert consultation, wherever they reside.

Earlier qualitative work with Somali women described mistrust and lack of confidence in providers in the US [20]. That could explain the relatively low utilization rates, which may reflect an unwillingness to visit providers. While our data suggest that a majority of Somali women trusted providers to provide quality care, approximately 16% reported feeling discriminated against. Importantly, reports of perceived discrimination were associated with reduced satisfaction in FGM/C-related care and lower rates of HSU. This suggests a pathway between discrimination and care seeking that needs additional exploration. The authors will be using additional data collected from this same study population to explore this relationship more deeply in a future publication. The possibility that FGM/C-affected women simply do not feel a need to seek care is also feasible; only about a quarter of participants reported having experienced adverse effects at the time of circumcision.

As important as it is for health care providers to be competent in providing care to FGM/C-affected women, increased awareness of FGM/C may have unintended consequences. In our efforts to provide services, we may suggest ‘iatrogenic pathology’ and risk making women who otherwise feel well to question their wellness and potentially feel ‘abnormal’. Lien and Schultz describe this as a possible moment of “epistemological change that occurs when deeply held attitudes change paradigmatically” and involves both a loss of pride and a feeling of shame that are painful” [41]. Providers should be cognizant of this potential when they approach FGM/C discussions with patients.

Our second aim was to describe individual-level patterns of and factors influencing HSU with respect to FGM/C. We were surprised to see perceived discrimination, refugee status, and FGM/C type lose significance in the final model. With respect to FGM/C type, a dose–response to FGM/C has been described, with more severe health complications associated with more severe types of cutting [42]. We hypothesized that FGM/C type would be associated with HSU. However, the only FGM/C-related variable to predict HSU was a woman’s recollection of physical and psychological effects at the time of the cutting. This suggests that more attention to women’s experiences versus the extent of cutting may assist in understanding HSU patterns. Most importantly, it highlights the importance of taking a thorough history to understand her personal experience with FGM/C. This is consistent with guidance in the WHO’s 2018 clinical handbook on FGM/C which suggests providers, after establishing a woman has a cutting history and is willing to discuss it further, inquire about a woman’s personal experience of FGM/C [43]. Understanding the nature of her recollection, if any, may provide valuable guidance about the type of therapies she may benefit from.

Other individual-level influences on HSU included ethnic group and acculturation status. Acculturated and Hypocultured respondents were associated with lower rates of HSU. Acculturated women may be more aware of and/or sensitive to the unique status of their genitals vis à vis other women and potentially less willing to access care because of that. Efforts to destigmatize a history of FGM/C may be an important strategy in building partnership with affected women. The “othering” phenomenon of the bodies of women of color generally, and of circumcised women of color in particular, require that clinicians establish respectful partnerships centered on safety, trust, and shared decision making [44].

Finally, we aimed to describe women’s experience of and ultimately, satisfaction with that care. In the WHO Quality of Care Framework, a patient’s experience of care is considered as important as the competence of the technical provision of care [25]. This emphasis on how women are made to feel during care experiences reflects a growing global acknowledgement that poor patient experience has the potential to negatively impact maternal and newborn outcomes [45]. The emphasis on patients’ experiences is reflected in the US, including by the Alliance for Innovation on Maternal Health which leads quality improvement in the US [46].

In this study, the patients’ experience of care countered common themes in qualitative research that suggest discriminatory and non-patient centered care is the norm [47,48]. In the current study, 83% of women denied perceptions of discrimination and most women had confidence in discussing FGM/C and trusted their providers. On the surface, this is reassuring and may reflect the positive impact of having a national expert in FGM/C in their proximity. Yet, 41% of women did not have confidence to advocate for their own FGM/C care and 21% did not trust their providers.

The differences in experience of care related to ethnicity, acculturation status, and education level reinforce the importance of patient-centered care. Somali Bantus, and women with Acculturated and Bicultural patterns of cultural involvement had poorer care experiences and lower odds of accessing services. These disparities reinforce the need for providers to be aware of the history of marginalization, and its impact on care, in an effort to seek equitable outcomes. Given the potential influence of Somali ethnicity on HSU, it is important that providers recognize the heterogeneity of Somali ethnic groups and that the poorest care experiences were reported by Somali-Bantus and women who reported a negative, initial physical or psychological sequalae at the time of cutting. Somali-Bantus’ poorer care experiences and access to care may be related to their history of marginalization and slavery in Somali culture. We do not know of other studies that have examined the relationship between the original FGM/C experience and future health seeking patterns; however, a separate examination of this phenomenon is currently underway using these data.

With respect to acculturation status, poorer care experiences among Acculturated and Bicultural women may reflect a ‘knowing’ of how they are different from uncut women. Our data may suggest that this knowing may be accompanied by shame, as discussed above, and may have negative impacts on communication with providers as a result. This reinforces how important it is for providers to de-stigmatize FGM/C and communicate accurate information about how they were affected. For example, many providers do not recognize that much of the clitoral body and its function remain intact following FGM/C and that many cut women do maintain sexual function [49]. If providers can share this information with women, it may counter feelings of shame or confusion about their history. In contrast, education was independently associated with an improved care experience: more educated women reported more comfort in discussing their FGM/C. A potentially important factor not captured in this study relates to the potential influences of spouses and family members who may act to enforce sociocultural or patriarchal norms affecting health service use. Research on this phenomenon may yield important additional insights.

Our data about care experience adds to the discussion around Respectful Maternity Care in the US. Studies find that pregnant Black women experience discrimination, poor treatment, and stereotyping based on their race, income, or age [50]. The body of research that describes poor care experiences of Black women suggests that what is preventing care of FGM/C-related women from being a more positive experience may not be the FGM/C, but socially constructed racial categorization. This suggests that national initiatives to address racial and ethnic disparities may benefit US-born Black women as well as diasporic Black women who are affected by FGM/C.

There are multiple, practical implications for patient care from this study. First, efforts need to be made to ensure clients can find providers with appropriate expertise or knowledge on how to obtain a consultation. Mechanisms for this have been developed in the US and providers are welcome to, by contacting the authors, explore joining the listserve where this informal community of practice exists. Recently, US federal grants to improve care of FGM/C-affected women have concluded, and additional provider tools have become available at https://endfgmnetwork.org/ (accessed on 31 March 2021).

With respect to FGM/C, the emphasis on women affected by Type III in the qualitative literature may discourage thorough exams, since providers may be looking for an obvious infibulation. Our data suggest that it is the history of a physical or psychological trauma that influences HSU—not the type of FGM/C. This should encourage all providers to resist dismissing the influence of Types I and II. Care must be taken to appropriately inquire about FGM/C during history-taking, with attention to women’s experience of the cutting itself, and complete a thorough genital perineal exam. There should be a palliative approach to address any health problems that women with FGM/C may experience. This includes a patient centered approach to openly discuss women’s goals, and to address any physical or psychological concerns related to FGM/C. Although a dose–response to FGM/C is commonly described, with more severe types being associated with more severe outcomes, our data suggest that more attention to women’s experience of the cutting itself may assist in understanding HSU patterns.

## 5. Strengths and Limitations

A limitation of this study is its reliance on self-reported FGM/C status. Given that this was a community-based study, it was not feasible to conduct pelvic exams to verify FGM/C status. However, the use of the pictorial aids (RAINBO guide) offers increased reliability, particularly in communities where Type III predominates. While this was not a random sample, it is the largest data set of Somali women collected in the US, successfully capturing the wide variation within the study population, including both Ethnic Somalis and Somali Bantus, patterns of cultural involvement and FGM/C type. Finally, the use of community-based participatory research methods throughout the design, implementation, and dissemination of this study arguably sustained the research during a time in the US where Somali communities were embroiled in a political climate of animus towards migrants, Muslims, and Somalis in particular.

## 6. Conclusions

As migration continues to mount globally, the effective and respectful care of migrant-specific women’s health concerns such as FGM/C, practices once bound to specific regions, will become increasingly pivotal in countries such as the US. While efforts are underway to end the practice globally, the large number of women with FGM/C worldwide makes ensuring access to quality care a continuing imperative. The findings of this study suggest that in the US achieving these aims have contextual complexity, given the effects of ongoing marginalization and acculturation. However, the clinical implications and insights provided may have broader impacts on advancing health equity for FGM/C-affected women.

## Figures and Tables

**Table 1:** Participant Characteristics.

	%	*n*	M	SD	Range
Age (years)			31.15	13.8	15–90
Ethnicity					
Ethnic Somali	73.7	631			
Somali Bantu	26.3	225			
Context of Emigration					
Involuntary migration (refugee)	78.3	611			
Voluntary migration	16	125			
U.S. Born	5.6	44			
Years in the U.S.			8.67	6.85	0–47
Education					
None	25.3	217			
Any	74.7	641			
Primary	16.1	138			
High School	42.2	362			
College	16.4	141			
Acculturation					
Bicultural	12.1	106			
Acculturated	36.1	316			
Traditional	33.3	291			
Hypocultural	18.5	162			
Household size					
1	8.3	71			
2	13.6	116			
3–5	39.3	335			
6+	38.8	331			
Annual Household Income					
<10,000 USD	41.4	247			
10–24,999 USD	38.8	231			
>25,000 USD	19.8	106			
Self-rated health					
Excellent	53.4	463			
Very Good	24.1	209			
Good	16.8	146			
Fair	4.9	43			
Poor	0.1	6			
Sought medical care for FGM/C-related health ever					
Yes	14.4	98			
No	85.6	589			
Insurance					
None	9.2	78			
Public	82.6	703			
Private	8.2	70			
FGM/C Status					
Uncircumcised	22.6	199			
Circumcised	77.4	680			
FGM/C Type *					
Type I	36.9	223			
Type II	23	139			
Type III	40.1	243			
Age of circumcision			7.09	2.27	0–15
Recall experiencing health problems at the time of circumcision					
Yes	26.7	143			
No	73.3	393			
Regret being circumcised					
Yes	25.7	173			
No	74.3	500			
Had choice whether to be circumcised					
Yes	10.1	69			
No	89.9	613			

* Using the RAINBO laminated guide [34], a visual aid arranged according to the three major categories of FGM/C as outlined by the World Health Organization’s FGM/C types; women were able to self-identify genitalia that most resembled their own FGM/C type.

**Table 2:** Access to care for FGM/C.

Dimension of Access	N (%)	FGM/C Health Service Use	Satisfaction in Care
		Unadjusted OR (95% CI)	Unadjusted OR (95% CI)
*Non-discrimination*			
Believe women with FGM/C discriminated against	136 (16.1)	1.89 (1.10–3.26) *	0.22 (0.13–0.37) *
*Physical Access*			
Did not have transportation	5 (0.7)	**	**
*Economic Access*			
Could not afford FGM/C care	6 (0.9)	**	**
Insurance would not approve	16 (2.4)	0.84 (0.19–3.78)	0.14 (0.05–0.41) *
*Informational Access*			
Know where to access health care for FGM/C concerns	490 (75.2)	1.79 (1.01–3.16) *	6.32 (3.82–10.45) *
*Health System Accessibility*			
Could not receive referral	24 (3.5)	1.59 (0.58–4.37)	0.10 (0.04–0.23) *
Could not find specialist	39 (5.7)	1.32 (0.57–3.09)	0.08 (0.04–0.15) *
Could not get appointment	2 (0.3)	**	**

* *p* < 0.05, ** Omitted due to small cell size.

**Table 3:** Unadjusted and Adjusted Odds Ratios of FGM/C Factors affecting FGM/C-related Health Service Use.

		Model A	Model B	Model C	Model D
		Macrostructural	FGM/C–Specific	Predisposing	Final
	Unadjusted	Adjusted (*n* = 649)	Adjusted (*n* = 553)	Adjusted (*n* = 636)	Adjusted (*n* = 564)
	OR (95% CI)	OR (95% CI)	OR (95% CI)	OR (95% CI)	OR (95% CI)
Age (covariate)	1.02 (1.00–1.03) *	1.02 (1.00–1.03) *	1.01 (0.99–1.03)	1.00 (0.98–1.02)	1.00 (0.98–1.02)
*Macrostructural/Contextual Factors*					
Perceived discrimination	1.71 (1.02–2.88) *	1.89 (1.10–3.26) *			1.67 (0.79–3.55)
Involuntary migration	5.26 (2.09–13.21) *	5.58 (2.19–14.2) *			1.48 (0.54–4.03)
*FGM/C Specific Factors*					
FGM/C Type					
Type I	Ref		Ref		
Type II	0.78 (0.39–1.58)		0.53 (0.25–1.16)		
Type III	2.11 (1.27–3.52) *		1.20 (0.66–2.20)		
Recall physical/mental sequela	4.42 (2.80–6.96) *		3.47 (2.01–5.99) *		3.09 (1.67–5.68) *
Regret being circumcised	2.18 (1.39–3.41) *		1.15 (0.66–1.99)		
Had choice whether or not circumcised	0.25 (0.08–0.80) *		0.26 (0.06–1.12)		
*Predisposing Factors*					
Education					
None	Ref				
Any	1.57 (0.92–2.68)				
Ethnicity					
Ethnic Somali	Ref			Ref	Ref
Somali Bantu	0.14 (0.06–0.36) *			0.15 (0.06–0.39) *	0.10 (0.02–0.44) *
Acculturation					
Traditional	Ref			Ref	Ref
Acculturated	0.31 (0.17–0.59) *			0.28 (0.15–0.54) *	0.39 (0.17–0.86) *
Bicultural	0.64 (0.33–1.24)			0.61 (0.31–1.20)	0.61 (0.25–1.47)
Hypocultural	0.29 (0.13–0.66) *			0.28 (0.12–0.65) *	0.27 (0.10–0.70) *
Cannot get preferred physician gender	5.84 (0.78–43.87)				
Years in US	0.98 (0.94–1.01)				
*Enabling Factors*					
Income					
<10,000 USD	Ref				
10–24,999 USD	1.57 (0.92–2.66)				
>25,000 USD	1.20 (0.61–2.36)				
Insurance (y/n)	2.37 (0.84–6.69)				
Household size					
1	Ref				Ref
2	1.16 (0.51–2.63)				1.25 (0.44–3.53)
3–5	0.66 (0.32–1.39)				0.69 (0.27–1.78)
6+	0.40 (0.20–0.88) *				0.48 (0.18–1.31)

* *p* < 0.05.

**Table 4:** Experiences in Care and Association to Satisfaction in Care.

	N (%)	Unadjusted
		OR (95%CI)
**Effective Communication**		
*Confidence in ability to advocate for the FGM/C–care desired*		
A great deal of confidence	475 (59.4)	Ref
Some confidence	192 (24.0)	0.33 (0.15–0.70) *
Very little confidence	70 (8.8)	0.06 (0.03–0.14) *
No confidence	63 (7.9)	0.02 (0.01–0.04) *
*Comfortable discussing FGM/C–related problems with provider*		
No	137 (20.9)	Ref
Yes	518 (79.1)	6.94 (4.17–11.56) *
**Respect and Dignity**		
*How much respect and dignity did your provider treat you with related to your circumcision*		
A great deal	490 (62.9)	Ref
A fair amount	200 (25.7)	0.26 (0.13–0.50) *
Not too much	37 (4.8)	0.05 (0.02–0.11) *
None at all	52 (6.7)	0.02 (0.01–0.04) *
*In your opinion, are women with FGM/C looked down on/ discriminated against by providers*		
No	709 (83.9)	Ref
Yes	136 (16.1)	0.22 (0.13–0.37) *
Autonomy		
*Have choice in where to receive care*		
A great deal of choice	546 (65.2)	Ref
Some choice	170 (20.3)	0.25 (0.14–0.45) *
Very little choice	65 (7.8)	0.13 (0.06–0.28) *
No choice	57 (6.8)	0.11 (0.05–0.23) *
*Have choice in provider*		
A great deal of choice	499 (59.8)	Ref
Some choice	185 (22.2)	0.17 (0.09–0.31) *
Very little choice	89 (10.7)	0.08 (0.04–0.17) *
No choice	62 (7.4)	0.12 (0.05–0.27) *
*Have choice in type of services receive*		
A great deal of choice	512 (61.1)	Ref
Some choice	186 (22.2)	0.20 (0.11–0.37) *
Very little choice	81 (9.7)	0.08 (0.04–0.17) *
No choice	59 (7.0)	0.12 (0.05–0.28) *
*Feel able to freely express desires regarding circumcision management with provider*		
No	194 (29.4)	Ref
Yes	467 (70.6)	6.18 (3.69–10.35) *
**Trust in Provider**		
*Trust provider to give good quality care in addressing FGM/C concerns*		
No	211 (26.4)	Ref
Yes	589 (73.6)	10.50 (6.28–17.55) *

* *p* < 0.05.

## Data Availability

The data presented in this study are available on request from the corresponding author. The data are not publicly available due to the sensitivity of the topic, the inclusion of minors, legal implications of self-reporting FGM/C of daughters given the current political, anti-immigrant climate in the US, as well as concerns around other potential unintended consequences to vulnerable communities.
